# Advancing precision medicine: Uncovering biomarkers and strategies to mitigate immune-related adverse events in immune checkpoint inhibitors therapy

**DOI:** 10.1016/j.toxrep.2025.102035

**Published:** 2025-04-24

**Authors:** K.L. Nityashree, P. Rachitha, Shilpa Hanchinmane, Vinay B. Raghavendra

**Affiliations:** a Christ University, Department of Psychology, Bangalore, India; b PG Department of Biotechnology, Teresian College, Siddarthanagar, Mysuru 570011, India; c PG Department of Biological Sciences, Jyoti Nivas College, Autonomous Koramangala, Bengaluru 560095, India

**Keywords:** Immunotherapy, Biomarkers, Precision medicine, Immuno-related adverse events, Immunosuppressants, Immunotoxicity

## Abstract

Immune-related adverse events (irAEs) can have a major influence on patient outcomes, but their usage is frequently confounded by immune checkpoint inhibitors (ICIs), which have revolutionized cancer treatment by increasing anti-tumor immunity. With a focus on immunological dysregulation and the resulting tissue-specific toxicities, this review clarifies the fundamental processes of irAEs. We look at contemporary clinical treatment techniques to lessen the impact of these adverse events, such as the application of immunosuppressants and patient monitoring procedures. Additionally, it is emphasized how future research is necessary to find predictive biomarkers that can forecast the development of irAEs, allowing for early intervention and individualized therapy methods. In order to improve the therapeutic index of ICIs, we also examine the crucial balance between optimizing anti-tumor activity and reducing immunotoxicity. This study aims to further the existing discussion on enhancing the safety and effectiveness of ICI medicines, which will eventually improve cancer patient care, by pointing out possible research avenues.

## Highlights

• Overview of Immune-Related Adverse Events (irAEs).
• Immunotoxicity in Cancer Therapies.
• Assessing and mitigating Immunotoxicity.
• Strategies for Ensuring Drug Safety in Immunotherapy.

## Graphical Abstract

**Figure:**
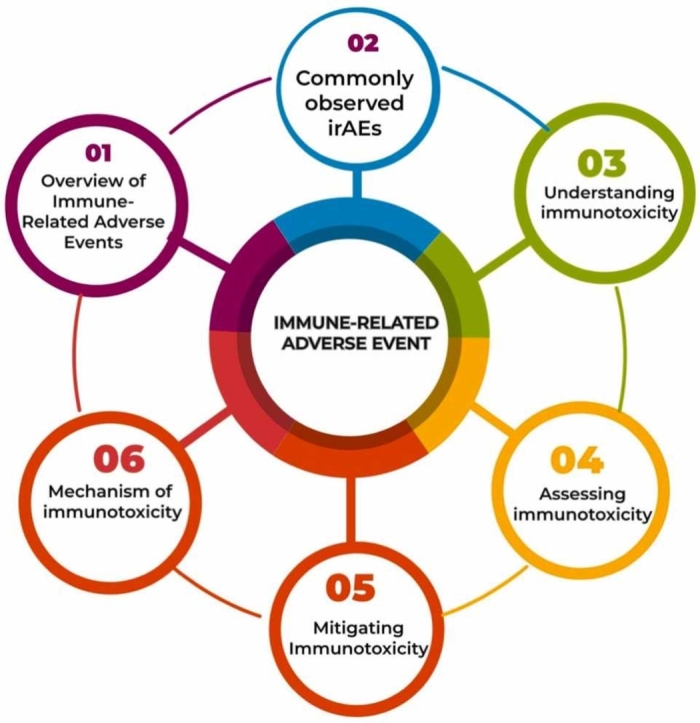

## 1 Introduction

Immunotherapies, in particular immune checkpoint inhibitors (ICIs), have revolutionized cancer therapy paradigms by improving survival for patients with previously incurable cancers. ICIs trigger an anti-tumor immune response by targeting inhibitory pathways in T-cells, including Cytotoxic T-Lymphocyte-Associated Protein 4 (CTLA-4), Programmed Cell Death Protein-1 (PD-1), and its ligand PD-L1. These treatments have shown impressive results in treating a variety of malignancies, such as renal cell carcinoma, non-small cell lung cancer, and melanoma [1]. However, immune-related adverse events (irAEs), which can cause serious damage when the immune system targets healthy tissues in addition to tumors, have emerged as a new difficulty with this advancement in oncologic therapy.

The spectrum of irAEs is broad and may affect nearly every organ system, manifesting in dermatologic, gastrointestinal, endocrine, and pulmonary complications, among others [2]. These events, which occur in approximately 15–60 % of patients depending on the ICI used and cancer type, can range from mild to life-threatening and may persist or even emerge after discontinuation of therapy [3]. For instance, recent real-world studies indicate that gastrointestinal irAEs such as colitis, endocrine disturbances like thyroiditis, and pneumonitis are among the most common and severe complications associated with ICIs [4].

The underlying mechanisms of irAEs are thought to involve the breakdown of immune tolerance, with activated T cells and other immune effectors targeting self-antigens. While steroids and other immunosuppressive agents have been used to manage these toxicities, long-term sequelae remain a concern, particularly for endocrine disorders that may require lifelong hormone replacement [5]. Given the increasing use of ICIs across cancer types and their expanding indications in earlier stages of disease, the management of irAEs has become a pressing issue in clinical oncology, requiring a multidisciplinary approach to care [6].

Future research is urgently needed to identify biomarkers that predict the onset of irAEs, allowing for early intervention and personalized treatment strategies. Additionally, exploring the balance between maximizing anti-tumor efficacy and minimizing immunotoxicity is critical to enhancing the therapeutic index of ICIs**.** This communication will discuss the mechanisms of irAEs, review the current clinical management approaches, and highlight potential research directions to improve the efficacy and safety of ICI therapy [7].

## 2 Immune-related adverse events (irAEs) in non-oncology contexts

Although mostly linked to cancer therapies, immune-related adverse events (irAEs) are becoming more widely recognized in non-oncological contexts, expanding the field of immunological research. The immune reconstitution inflammatory syndrome (IRIS), which is seen in a number of immunocompromised conditions, is one prominent symptom. For example, when immunosuppressive medication is reduced or stopped, solid organ transplant recipients may experience IRIS, which can result in unmasked infections including, TB and CMV. Similar to this, IRIS can occur in neutropenic patients recuperating from low neutrophil counts because of the abrupt return of immune function, which could worsen latent infections [8]**.**

The immune system changes from a repressed condition during pregnancy to a pro-inflammatory state after delivery, which can reactivate illnesses including, viral hepatitis and cryptococcosis. Postpartum people are particularly at risk for IRIS. Patients who stop using tumor necrosis factor antagonists, which are used to treat chronic inflammatory disorders, may also have IRIS because of reduced granuloma formation that results in the reactivation of latent infections [9].

One example of an irAE that happens outside of oncology is cytokine release syndrome (CRS). A feedback loop of increasing inflammation is created when a significant number of white blood cells produce inflammatory cytokines, leading to this hyperinflammatory state. Numerous infectious and non-infectious diseases have been linked to CRS, such as severe acute respiratory syndrome coronavirus 2 (SARS-CoV-2) infections, in which patients with severe COVID-19 show increased levels of cytokines like ferritin and IL-6, which can lead to heart problems and acute respiratory distress syndrome [10].

IrAEs with substantial morbidity include severe cutaneous adverse reactions (SCARs), which include diseases such as toxic epidermal necrolysis (TEN) and Stevens-Johnson syndrome (SJS). Although frequently caused by drugs, SCARs have also been connected to infections, such as the reactivation of Mycoplasma pneumoniae and herpesviruses, highlighting the intricate relationship between immune responses and infections in these side effects [11].

Utilizing new technology is necessary to address these non-oncology irAEs. Early treatments are made possible by artificial intelligence (AI), which can evaluate large datasets to identify those who are at risk for irAEs. Complex immunological interactions may be modeled using systems biology techniques, which shed light on the processes behind irAEs. By combining proteomic, transcriptomic, and genomic data, multi-omics technologies can find biomarkers for irAEs and open the door to individualized treatment plans. When combined, these technologies have the potential to improve patient outcomes by clarifying the pathophysiology of irAEs in a variety of therapeutic contexts [12].

## 3 Overview of immune-related adverse events (irAEs)

A wide range of autoimmune-like reactions brought on by immune checkpoint inhibitors (ICIs), which have significantly improved patient outcomes for patients with a variety of cancers, are referred to as irAEs. ICIs improve the immune system's capacity to identify and combat tumor cells. Examples of these include those that target CTLA-4, PD-1, and PD-L1. Off-target immune activation and consequent harm to healthy tissues result from their disruption of the delicate balance of immunological tolerance [13]. IrAEs, which can impact almost any organ system and manifest in a range of severity from mild, manageable symptoms to life-threatening illnesses, are caused by this breakdown in self-tolerance [14].

Multiple organ systems may be impacted by the wide range of clinical symptoms of irAEs. Among the most frequent adverse drug responses (irAEs), dermatologic events like rash, vitiligo, and pruritus frequently manifest early in the course of treatment. If not treated right once, serious gastrointestinal adverse events, especially colitis, might result with complications such as perforation [15]**.** Permanent hormone shortages resulting from endocrine toxicities, such as thyroiditis, hypophysitis, and adrenal insufficiency, necessitate lifelong hormone replacement therapy [16]**.** Furthermore, although less common, pulmonary toxicities, including pneumonitis are significant side effects that can cause respiratory failure and necessitate vigorous immunosuppressive treatment [17]**.**

One of the major challenges in managing irAEs is their unpredictable timing. While some events may occur early in the course of treatment, others can emerge weeks to months after therapy initiation, and in some cases, irAEs have been observed long after the cessation of ICI treatment [18]**.** This delayed onset complicates patient monitoring and requires long-term vigilance, even in patients who have discontinued treatment. Additionally, the severity and reversibility of irAEs can vary; while some toxicities, such as mild skin rashes, are reversible with corticosteroids or other immunosuppressive treatments, others, like endocrine disorders, may result in permanent organ damage [19]**.**

**Fig. 1:**
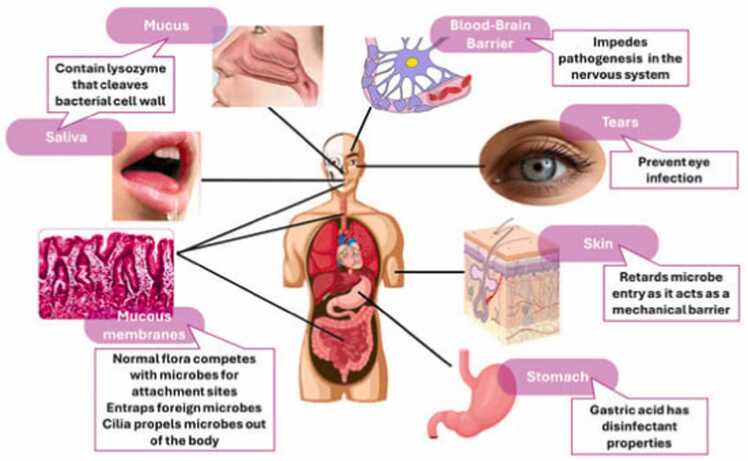
An overview of immune-related adverse events [23].

The underlying mechanisms of irAEs are still being explored, but they are thought to involve the activation of autoreactive T-cells and a loss of peripheral immune tolerance. This loss of tolerance may be exacerbated by genetic predispositions, environmental factors, and the tumor microenvironment [20]**.** Furthermore, the occurrence of irAEs may correlate with better anti-tumor responses, suggesting that a heightened immune response against cancer cells could also increase the risk of autoimmune damage. This complex interplay highlights the need for more research to identify biomarkers that can predict the onset of irAEs, allowing for more tailored and preemptive interventions [21]**.** Given the increasing use of ICIs across a wide range of cancers, including their expanding indications in early-stage cancers and combination therapies, the incidence of irAEs is expected to rise. This underscores the importance of multidisciplinary approaches to managing irAEs, involving oncologists, immunologists, and other specialists to ensure timely diagnosis, appropriate treatment, and long-term monitoring [22]**.** Future research should focus on elucidating the mechanisms underlying irAEs, developing predictive biomarkers, and exploring novel strategies to mitigate these toxicities without compromising the efficacy of cancer immunotherapy (Fig.1).

### 3.1 Commonly observed irAEs

This study aimed to investigate the correlation between the occurrence of immune-related adverse events (irAEs) and the clinical response to nivolumab (NIVO) or nivolumab plus ipilimumab (NIVO+IPI) in patients with advanced solid tumors. The researchers conducted a systematic review and meta-analysis of clinical trials that reported the incidence of irAEs and clinical outcomes in patients treated with NIVO or NIVO+IPI. They included studies that reported data on at least one of the following irAE categories: skin, gastrointestinal, endocrine, hepatic, pulmonary, or renal. The pooled incidence of any grade irAE was 80 % with NIVO and 91 % with NIVO+IPI. The most common irAEs with NIVO were pruritus (itching), diarrhea, and rash. The most common irAEs with NIVO+IPI were diarrhea, pruritus, and rash. The overall response rate (ORR) was significantly higher in patients who experienced irAEs than those who did not. The ORR was positively correlated with the incidence of skin, gastrointestinal, and endocrine irAEs with both NIVO and NIVO+IPI. This study demonstrates a strong correlation between the occurrence of irAEs and the clinical response to NIVO and NIVO+IPI in patients with advanced solid tumors. These findings suggest that irAEs may be a biomarker of treatment response and that close monitoring for irAEs is essential in patients receiving these therapies [24].

The most common irAEs in NSCLC are skin reactions and gastrointestinal (GI) problems. The management of irAEs typically involves dose reduction, interruption, or discontinuation of ICI therapy, along with supportive care. In some cases, corticosteroids may be used to suppress the immune response. Patients with NSCLC can experience multiorgan irAEs, affecting multiple organ systems simultaneously. These can be more complex to manage and may require a multidisciplinary approach. Rechallenge with ICIs after resolution of irAEs is possible in some cases, but it should be approached with caution and careful monitoring. This review highlights the importance of recognizing and managing irAEs in patients with NSCLC who are treated with ICIs. Early identification and prompt intervention can help to minimize the severity and duration of irAEs and improve patient outcomes [25].

### 3.2 Among the various irAEs, some of the most commonly reported include

Immunotherapy is a promising treatment approach for various cancers, but it can also lead to a range of side effects known as irAEs. These occur when the immune system, activated by immunotherapy, starts attacking healthy tissues.

#### 3.2.1 Dermatologic reactions

**Skin rashes** can range from mild to severe and may appear as a rash, itching, or inflammation. **Pruritus:** This is a medical term for severe itching. **Dermatitis:** This is inflammation of the skin [26], [27].

#### 3.2.2 Gastrointestinal issues

**Colitis:** This is colon inflammation, leading to symptoms like diarrhea, abdominal pain, and rectal bleeding. It can be a severe condition requiring urgent medical attention [28].

#### 3.2.3 Endocrinopathies

**Thyroiditis:** This is inflammation of the thyroid gland, which can lead to hypothyroidism (underactive thyroid) or hyperthyroidism (overactive thyroid). **Adrenal insufficiency:** This occurs when the adrenal glands don't produce enough hormones, leading to fatigue, weight loss, and low blood pressure [29].

#### 3.2.4 Pneumonitis

**Lung inflammation:** This can cause shortness of breath, cough, and chest pain. It can be a serious condition requiring immediate medical intervention [30].

The management of irAEs presents several challenges due to their unpredictable nature and the complexity of immune responses.

## 4 Delayed onset and heterogeneity

One of the primary challenges is the delayed onset of irAEs, which can occur weeks to months after the commencement of immunotherapy. Clinicians must maintain a high index of suspicion to recognize these events, especially since they can mimic disease progression or other unrelated medical conditions. The heterogeneity of symptoms further complicates diagnosis. For instance, gastrointestinal symptoms may overlap with common side effects of chemotherapy, making it difficult to distinguish between irAE and other etiologies.The management of irAEs associated with immune checkpoint inhibitors (ICIs) is notably complex, due to their delayed and often unpredictable onset. These events may occur weeks or even months after initiating immunotherapy, posing a diagnostic challenge. Clinicians must be vigilant, as irAEs may mimic disease progression or other conditions, complicating early recognition and treatment [31].

## 5 Management decisions

The management of irAEs often involves corticosteroids or immunosuppressive agents to mitigate the immune response. However, this raises a significant concern: the potential impact on the therapeutic efficacy of the immunotherapy. While corticosteroids are effective in addressing inflammation, their use may compromise the overall immunotherapeutic response, leading to poorer outcomes in some patients. Another major challenge involves the heterogeneity of symptoms across different organ systems, such as gastrointestinal, hepatic, or dermatologic, which can overlap with other treatment-related effects. The difficulty in distinguishing irAEs from similar conditions emphasizes the need for clear diagnostic guidelines [32]. Management often involves immunosuppressive treatments like corticosteroids. While these are generally effective in reducing inflammation and preventing organ damage, there is concern that they may dampen the immune response crucial for tumor control. This trade-off is a critical consideration, as inappropriate or delayed management could lead to adverse patient outcomes [33].

### 5.1 Understanding immunotoxicity

Immunotoxicity refers to the adverse effects on the immune system caused by pharmaceutical agents, which can lead to immune dysregulation, hypersensitivity, or immunosuppression. In the context of cancer therapies, understanding immunotoxicity is essential to improve drug safety and patient outcomes [34].

### 5.2 Immune dysregulation

This can lead to an overactive immune response, resulting in autoimmune-like conditions where the body attacks its own tissues. **Hypersensitivity:** This refers to an exaggerated immune response to a specific antigen, leading to allergic reactions. **Immunosuppression:** This occurs when the immune system is weakened, making the body more susceptible to infections [35].

### 5.3 Immunotoxicity in cancer therapies

Cancer therapies, especially immunotherapies, can have a significant impact on the immune system. **Chemotherapy:** Can suppress bone marrow function, leading to decreased production of immune cells. May damage the thymus, an organ crucial for T-cell development. Can cause oxidative stress, which can damage immune cells. **Immunotherapy:** While designed to boost the immune system, can sometimes lead to unintended consequences like autoimmune reactions. Immune checkpoint inhibitors, a type of immunotherapy, can release the brakes on the immune system, leading to a heightened immune response that can sometimes target healthy tissues [36].

### 5.4 Assessing immunotoxicity

To assess immunotoxicity, researchers and clinicians use a variety of methods:

**Laboratory Tests:** Complete Blood Count (CBC) to monitor white blood cell counts., Liver function tests to assess potential liver damage, Kidney function tests to assess potential kidney damage, Immunophenotyping to analyze the number and function of different immune cell types. **Clinical Evaluation:** Monitoring for signs and symptoms of autoimmune diseases, such as rashes, joint pain, or fatigue, tracking the occurrence of infections [37].

### 5.5 Mitigating immunotoxicity

**Careful Patient Selection:** Identifying patients who are more likely to benefit from immunotherapy and less likely to experience severe side effects, **Dose Adjustment:** Adjusting the dosage of the drug to minimize side effects, **Combination Therapies:** Combining immunotherapy with other drugs to reduce the risk of severe side effects., **Immunomodulatory Therapies:** Using medications to suppress the immune system when necessary [38].

**Mechanisms of Immunotoxicity**

Immunotoxicity refers to the adverse effects on the immune system caused by pharmaceutical agents, which can lead to immune dysregulation, hypersensitivity, or immunosuppression. This is particularly relevant in the context of cancer therapies, especially with the rise of immunotherapies.

### 5.6 Immunosuppression

**Fig. 2:**
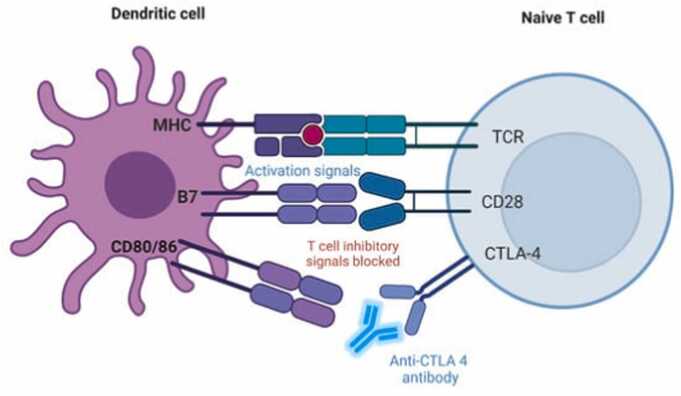
Figure showing CTLA-4 checkpoint inhibition mechanism [41].

**Fig. 3:**
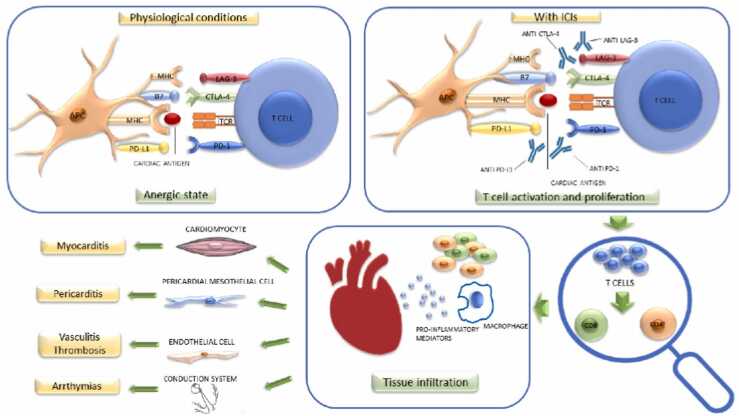
Figure showing Potential pathogenetic mechanism of cardiotoxicity induced by immune checkpoint inhibitors (ICIs) [42].

**Direct cytotoxicity**: Some drugs, such as certain chemotherapeutic agents, can directly kill immune cells, leading to a decreased immune response. Interference with immune cell signaling: Other drugs can interfere with the signaling pathways that regulate immune cell function, leading to impaired immune responses. **Bone marrow suppression:** Some drugs can suppress bone marrow function, leading to decreased production of immune cells, including lymphocytes and myeloid cells. **Hypersensitivity Reactions:** Type I hypersensitivity: This involves IgE-mediated reactions, such as allergic reactions, which can manifest as skin rashes, respiratory symptoms, or anaphylaxis. Type II hypersensitivity: This involves antibody-mediated cytotoxicity, where antibodies target specific cell surface antigens, leading to cell damage. Type III hypersensitivity: This involves immune complex-mediated reactions, where immune complexes deposit in tissues, leading to inflammation and tissue damage. Type IV hypersensitivity: This involves cell-mediated immunity, where T-cells are activated and attack specific tissues, leading to delayed-type hypersensitivity reactions. **Autoimmune Responses:** Molecular mimicry: Some drugs may share structural similarities with self-antigens, leading to the production of autoantibodies that target self-tissues. **Release of self-antigens:** Drug-induced tissue damage can release self-antigens, which can trigger an autoimmune response. Dysregulation of immune checkpoints: Immunotherapy drugs, such as checkpoint inhibitors, can disrupt the balance of immune regulation, leading to autoimmune side effects [39], [40] (Fig. 2, Fig. 3).

### 5.7 Strategies for ensuring drug safety in immunotherapy

The advent of immunotherapy has revolutionized cancer treatment, offering promising outcomes for various malignancies. However, ensuring drug safety remains a significant challenge due to the complex irAEs associated with these therapies. Effective strategies to mitigate risks involve robust monitoring systems, personalized treatment approaches, and integration of advanced technologies for real-time assessment [43], [44].

## 6 Risk assessment and patient monitoring

Implementing comprehensive risk assessment frameworks before and during treatment is crucial. These strategies include pre-treatment screening for autoimmune conditions and continuous monitoring of biomarkers predictive of irAEs, like cytokine profiles. Real-time data monitoring facilitates early detection and management of complications, minimizing long-term patient harm [45], [46].

**Personalized Medicine Approaches:** Personalization in immunotherapy is essential, as genetic and immunological variations impact safety profiles. Advances in biomarker research have allowed more accurate patient stratification, enhancing therapeutic outcomes while reducing adverse effects. Tailored regimens based on genetic analyses and immune profiling can guide dosing and mitigate the risks associated with immune checkpoint inhibitors and CAR-T therapies [47], [48].

**Technological Integration:** Artificial intelligence (AI) and Machine Learning (ML) are becoming integral in predicting and managing irAEs. These technologies analyze large datasets to identify patterns associated with safety risks, enabling proactive management strategies. Additionally, AI tools assist in optimizing treatment plans, thus improving overall safety [49], [50], [51].

**Fig. 4:**
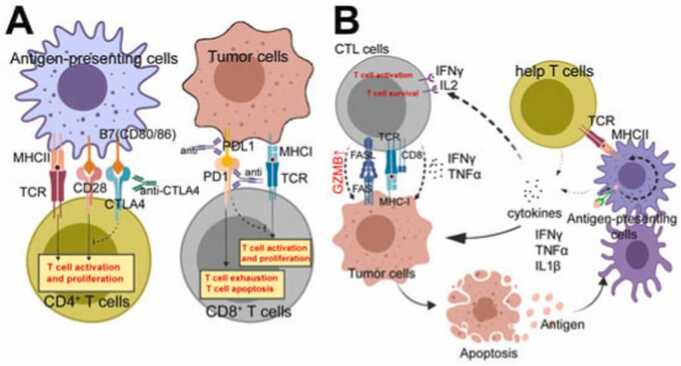
Figure showing Immunocytes in Tumor Immunotherapy [55].

**Collaboration and Regulatory Frameworks:** Collaboration between stakeholders, including pharmaceutical companies, healthcare providers, and regulatory bodies, is critical to ensure the safe deployment of immunotherapeutic agents. Updating regulatory guidelines to reflect emerging safety concerns and integrating patient safety into drug approval processes will promote more secure use of these therapies [52], [53], [54] (Fig.4).

## 7 Emerging technologies like AI, systems biology, or Multi-omics approaches

The development of therapeutic approaches is constrained by a number of information gaps that still exist despite significant progress in our understanding of immune system dynamics. Clarifying the specific molecular pathways via which environmental stresses affect immune regulation is one significant gap. Although immunological dysregulation and stress exposure have been connected in research, little is known about the cytokine profiles and T-cell fatigue routes. To fill these gaps, emerging technologies like systems biology and AI provide intriguing answers. Complex immune response patterns may be analysed by AI-driven models, providing predictive insights into immune cell behavior and cytokine interactions. Additionally, systems biology builds comprehensive models that show the dynamic interaction between immune regulation and environmental stresses by integrating data at several scales. In order to improve diagnosis accuracy and treatment plans, multi-omics techniques that integrate genomes, transcriptomics, and proteomics have also demonstrated promise in finding biomarkers for immunological dysfunction brought on by stress [56].

Furthermore, even though the gut microbiota is known to have a significant role in immunological homeostasis, further research is needed to identify the precise microbial strains and metabolite profiles that boost immune resilience. Finding important microbial strains that alter immunological pathways can be sped up by utilizing multi-omics techniques and AI-based microbial network analysis. The effect of ongoing psychosocial stress on immunological ageing is another topic that needs research. The underlying epigenetic changes driving this impact are still poorly understood, despite indications of accelerated immunological ageing under chronic stress circumstances. Frameworks from systems biology that use epigenomic data can provide information about these changes and suggest possible areas for action. Combining genomes, transcriptomics, and proteomics, multi-omics techniques have also demonstrated promise in finding biomarkers for immunological dysfunction brought on by stress, improving treatment approaches and diagnostic accuracy [57].

Additionally, there are still unanswered questions about how long-term immunological memory reacts to newly developing infectious illnesses. Although vaccinations offer protection, further research is needed to understand how memory B-cells target conserved viral epitopes for long-lasting immunity. The discovery of memory cell dynamics in developing infections can be facilitated by AI algorithms that are intended to analyse B-cell receptor repertoires. To advance immunological research and provide focused therapies, these gaps must be filled. Key insights into these fields have been emphasised by recent studies. The important function of gut microbiota in regulating inflammation and immunity, especially through metabolites generated from microbes [58].

Insightful information about immunological ageing, pointing to epigenetic modifications and inflammatory markers as major causes. Furthermore, memory B-cells' function in long-term immune responses, emphasising their capacity for persistent protection against viral infections. To guide future research directions and clinical advancements, more research is required into the intricate interactions between environmental stressors, gut microbiota, immune ageing, and long-term immune memory. This is highlighted by developments in multi-omics approaches, AI integration, and systems biology frameworks [59].

## 8 Future directions in managing irAEs and improving drug safety

The rapid rise in the use of ICIs for cancer treatment has made the management of irAEs increasingly crucial. ICIs work by enhancing the immune response against cancer cells but can also trigger adverse effects as the immune system targets healthy tissues. Commonly affected systems include gastrointestinal, hepatic, dermatologic, and endocrine organs, and the severity of irAEs can range widely depending on the specific agent, dose, and patient factors. Understanding and improving the management of irAEs has become essential to maximize the therapeutic benefits of ICIs while mitigating risks [60], [61], [62].

### 8.1 Risk stratification and biomarker development

A key direction in irAE management involves risk stratification and the identification of biomarkers predictive of irAE development. Biomarkers could enable more precise predictions of which patients are likely to experience irAEs, thus allowing for tailored treatment strategies that balance efficacy and safety. Research is ongoing to identify genetic and phenotypic markers that may indicate irAE susceptibility. For example, genetic variants related to immune regulation have shown promise as potential indicators, which could guide patient selection and dosing protocols to reduce irAE incidence [63], [64], [65].

### 8.2 Personalized management strategies and protocols

With irAEs affecting a wide range of organs and presenting variable severity, there is a strong call for individualized management protocols. Many institutions are developing structured, multidisciplinary approaches to address irAEs effectively. This includes integrating specialists such as oncologists, immunologists, and organ-specific experts to monitor and manage these events across departments. Moreover, standardized institutional protocols, including early detection strategies and predefined response algorithms, are being recommended to ensure timely and appropriate management of irAEs. Studies indicate that these protocols not only improve patient outcomes but also minimize the interruptions in cancer treatment, thus preserving overall therapy effectiveness [66], [67], [68].

### 8.3 Reducing dependence on high-dose steroids

Current standard treatment for severe irAEs involves high-dose corticosteroids, which carry their own risk of side effects and can impact the anti-cancer effectiveness of ICIs. Emerging approaches aim to reduce dependence on steroids by using targeted immunosuppressants such as TNF inhibitors, which may manage irAEs with fewer systemic effects. These strategies, supported by recent research, focus on addressing the inflammatory response associated with irAEs in a way that is less disruptive to the immune system’s anti-cancer functions [69], [70].

### 8.4 Long-term monitoring and chronic irAE management

As irAEs can persist long after discontinuing ICIs, long-term monitoring protocols are needed to manage chronic irAEs. This includes follow-up assessments to detect late-onset adverse events, particularly in patients with high-risk profiles. Enhanced surveillance, possibly utilizing telemedicine, allows for regular patient check-ins to catch delayed irAEs before they escalate. Continuous patient education on recognizing symptoms and seeking prompt medical attention remains a cornerstone of long-term irAE management strategies [71], [72], [73].

## 9 Conclusion

The emergence of immunotherapy as a potent weapon against cancer has revolutionized the landscape of oncology. However, its potential to elicit immune-related adverse events (irAEs) and immunotoxicity presents a significant challenge. A comprehensive understanding of these adverse effects is crucial to mitigate risks and optimize treatment outcomes. To address this challenge, a multi-faceted approach is necessary. Rigorous monitoring protocols, including regular laboratory tests and imaging studies, are essential for early detection and intervention. A multidisciplinary team approach, involving oncologists, immunologists, and other specialists, can facilitate timely and effective management of irAEs. Additionally, ongoing research into biomarkers and the gut microbiome holds promise for personalized medicine and novel therapeutic strategies. By prioritizing patient safety, investing in research, and fostering collaboration among healthcare providers, we can harness the full potential of immunotherapy while minimizing its adverse effects. Ultimately, the goal is to strike a balance between efficacy and safety, ensuring that patients can benefit from these groundbreaking therapies without compromising their quality of life.

Even while our understanding of immune system dynamics has advanced significantly, there are still a number of information gaps that restrict the development of new treatment approaches. Understanding the molecular processes by which environmental stresses affect immune regulation is one important knowledge gap. Although studies have connected immunological dysregulation and stress exposure, little is known about the cytokine profiles and T-cell fatigue routes. Although the gut microbiota is known to have a significant role in immunological homeostasis, further research is needed to identify the precise microbial strains and metabolite profiles that boost immune resilience. Although accelerated immunological aging has been associated with chronic psychosocial stress, little is known about the underlying epigenetic changes that mediate this impact. Further research is required to understand how long-term immunological memory reacts to newly developing infectious illnesses. Recent research has emphasized the potential of memory B-cells in maintaining immunity against viral infections, the negative effects of chronic stress on immune functioning, the importance of gut microbiota in regulating immunity and inflammation, and the important roles played by inflammatory indicators and epigenetic modifications. In order to guide future research paths and clinical breakthroughs, our findings highlight the need for more study into the intricate interactions among gut microbiota, immunological aging, environmental stresses, and long-term immune memory.

## CRediT authorship contribution statement

**Vinay B. Raghavendra:** Writing – review & editing, Formal analysis. **Shilpa Rachitha:** Resources, Methodology. **P. Rachitha:** Writing – review & editing, Methodology. **K.L. Nityashree:** Writing – review & editing, Writing – original draft.

## Declaration of Competing Interest

The authors declare that they have no known competing financial interests or personal relationships that could have appeared to influence the work reported in this paper.

## Data Availability

Data will be made available on request.
